# Cutaneous involvement in diffuse large B-cell lymphoma

**DOI:** 10.1093/omcr/omae069

**Published:** 2024-06-07

**Authors:** Seigi Oshima, Satoshi Takahashi, Toshiyuki Kitano

**Affiliations:** Department of Hematology, Medical Research Institute KITANO HOSPITAL, 2-4-20 Ohgimachi, Kita-ku, Osaka 530-8480, Japan; Department of Hematology, Medical Research Institute KITANO HOSPITAL, 2-4-20 Ohgimachi, Kita-ku, Osaka 530-8480, Japan; Department of Hematology, Medical Research Institute KITANO HOSPITAL, 2-4-20 Ohgimachi, Kita-ku, Osaka 530-8480, Japan

**Figure 1:**
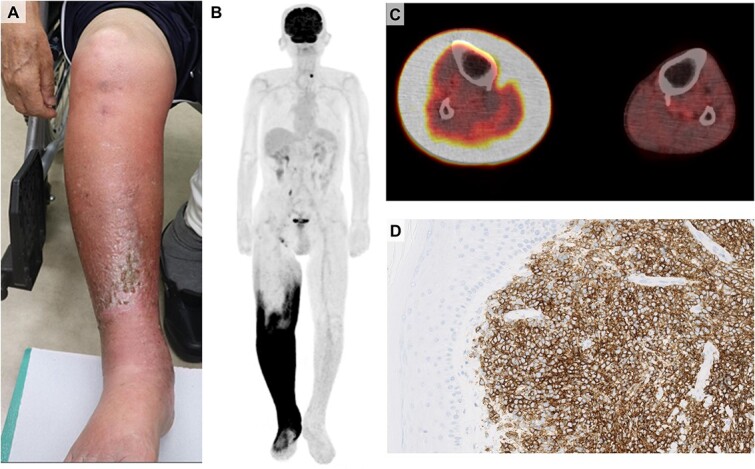
(**A**) The skin lesion of the right leg. (**B** and **C**) The findings of FDG-PET. Markedly elevated FDG uptake was observed specifically in the dermis and subcutaneous tissue of the right leg, sparing the adjacent muscle and bone. (**D**) Histopathological examination of the right leg biopsy samples. Immunohistochemical staining for CD20. Aggressive infiltration of lymphoma cells was observed mostly in the dermis and subcutaneous tissue.

An 85-year-old man presented with swelling and pain in his right lower leg, resembling cellulitis. One year earlier, he had been diagnosed with diffuse large B-cell lymphoma, not otherwise specified (DLBCL-NOS, Ann Arbor Stage IVB). Following seven cycles of R-CHOP therapy (rituximab, cyclophosphamide, doxorubicin, vincristine, and prednisone), the patient achieved a partial response except for the right adrenal gland, and chemotherapy was discontinued due to chemotherapy-induced peripheral neuropathy. Six months later, he developed swelling and pain in his right leg. On examination, no palpable lymph nodes were detected, but swelling with bullous lesions was observed in the right lower leg (Fig. 1A). Despite weeks of empiric antibiotic treatment, symptoms worsened. Laboratory findings revealed an elevated lactate dehydrogenase level of 403 U/L and a soluble interleukin-2 receptor level of 2278 U/mL, with no leukocytosis. Remarkably, fluorodeoxyglucose-positron emission tomography (FDG-PET) not only identified several small cervical and right inguinal lymph node lesions but also demonstrated high FDG uptake localized specifically to the subcutaneous tissue of the right leg, sparing the adjacent muscle and bone (Fig. 1B and C). A skin biopsy of the leg revealed aggressive infiltration of CD20-positive large-sized lymphocytes exclusively in the dermis and subcutaneous lesion, almost completely replacing normal tissue (Fig. 1D). These lymphocytes were positive for BCL-6, CD79a, MUM-1, and negative for CD3, CD10, and cyclin D1, consistent with the previous lymphoma biopsy. Cutaneous involvement of lymphoma can be categorized as primary or secondary [1]. Primary cutaneous lymphoma originates solely in the skin without any other lesions and has a better prognosis [2]. Secondary skin involvement is defined as the infiltration of systemic lymphoma to the cutaneous lesions. Among secondary lymphomas, approximately two-thirds are of B-cell origin, with DLBCL being the most prevalent histological type [2]. The patient underwent treatment with a salvage regimen including rituximab and polatuzumab vedotin, but died seven days after treatment due to tumor lysis syndrome. Clinicians should be aware of the cutaneous involvement of lymphoma when presented with skin lesions resembling cellulitis that do not respond to antibiotic treatment.
